# Dichlorido(η^6^-*p*-cymene)(4-fluoro­aniline-κ*N*)ruthenium(II)

**DOI:** 10.1107/S1600536810051962

**Published:** 2010-12-18

**Authors:** Richard E. Sykora, Andrew G. Harris, Jason W. Clements, Norris W. Hoffman

**Affiliations:** a Department of Chemistry, University of South Alabama, Mobile, AL 36688-0002, USA

## Abstract

The title compound, [RuCl_2_(C_10_H_14_)(C_6_H_6_FN)], a pseudo-octa­hedral *d* ^6^ complex, has the expected piano-stool geometry around the Ru(II) atom. The fluoro­aniline ring forms a dihedral angle of 19.3 (2)° with the *p*-cymene ring. In the crystal, two mol­ecules form an inversion dimer *via* a pair of N—H⋯Cl hydrogen bonds. Weak inter­molecular C—H⋯Cl inter­actions involving the *p*-cymene ring consolidate the crystal packing.

## Related literature

For applications of (η^6^-*p*-cymene)Ru(II) dihalides in organic synthesis, see: Boutadla *et al.* (2010 ▶). For studies of (η^6^-arene)Ru(II) dihalides in bioinorganic chemistry, see: den Heeten *et al.* (2010 ▶). For anti-tumor medical applications of (η^6^-arene)Ru(II) systems, see: Hanif *et al.* (2010 ▶). For conversion of [(η^6^-*p*-cymene)RuCl_2_]_2_ with two molar equivalents of neutral unidentate nitro­gen ligands into monomeric pseudo-octa­hedral piano-stool complexes of general formula (η^6^-*p*-cymene)Ru(N-ligand)Cl_2_, see: Burrell & Steedman (1997 ▶); Govindaswamy & Kollipara (2006 ▶); Begley *et al.* (1991 ▶). For crystal structures of Ni-triad complexes of 4-fluoro­aniline, see: Randell *et al.* (2006 ▶); Fawcett *et al.* (2005 ▶); Padmanabhan *et al.* (1985 ▶). For applications of ^19^F-NMR reporter moieties in monitoring ligand-substitution equilibria, see: Hoffman *et al.* (2009 ▶); Carter *et al.* (2004 ▶).
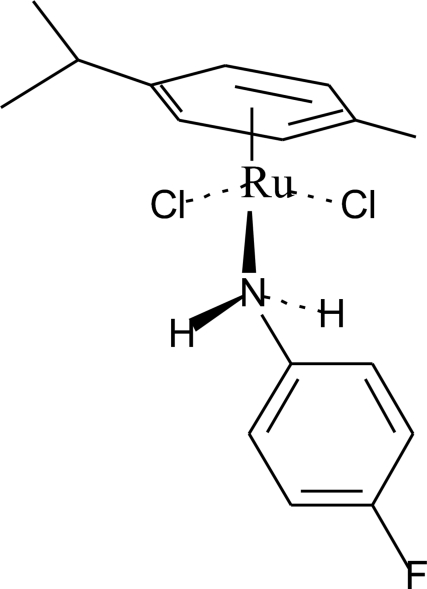


## Experimental

### 

#### Crystal data

[RuCl_2_(C_10_H_14_)(C_6_H_6_FN)]
*M* *_r_* = 417.30
Monoclinic, 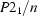

*a* = 8.6492 (9) Å
*b* = 12.2458 (13) Å
*c* = 15.6471 (16) Å
β = 93.271 (8)°
*V* = 1654.6 (3) Å^3^
*Z* = 4
Mo *K*α radiation
μ = 1.27 mm^−1^
*T* = 290 K
0.26 × 0.25 × 0.20 mm

#### Data collection

Enraf–Nonius CAD-4 diffractometer
Absorption correction: ψ scan (North *et al.*, 1968 ▶) *T*_min_ = 0.635, *T*_max_ = 0.779
3234 measured reflections
3027 independent reflections
2284 reflections with *I* > 2σ(*I*)
*R*_int_ = 0.048
3 standard reflections every 120 min  intensity decay: none

#### Refinement

*R*[*F* ^2^ > 2σ(*F* ^2^)] = 0.034
*wR*(*F* ^2^) = 0.090
*S* = 1.00
3027 reflections
193 parameters
H-atom parameters constrained
Δρ_max_ = 0.57 e Å^−3^
Δρ_min_ = −0.67 e Å^−3^

### 

Data collection: *CAD-4-PC* (Enraf–Nonius, 1993 ▶); cell refinement: *CAD-4-PC*; data reduction: *XCAD-4PC* (Harms & Wocadlo, 1995) ▶; program(s) used to solve structure: *SHELXS97* (Sheldrick, 2008 ▶); program(s) used to refine structure: *SHELXL97* (Sheldrick, 2008 ▶); molecular graphics: *OLEX2* (Dolomanov *et al.*, 2009 ▶); software used to prepare material for publication: *publCIF* (Westrip, 2010 ▶).

## Supplementary Material

Crystal structure: contains datablocks I, global. DOI: 10.1107/S1600536810051962/is2638sup1.cif

Structure factors: contains datablocks I. DOI: 10.1107/S1600536810051962/is2638Isup2.hkl

Additional supplementary materials:  crystallographic information; 3D view; checkCIF report

## Figures and Tables

**Table 1:** Hydrogen-bond geometry (Å, °)

*D*—H⋯*A*	*D*—H	H⋯*A*	*D*⋯*A*	*D*—H⋯*A*
N1—H1*B*⋯Cl2^i^	0.90	2.39	3.225 (3)	154
C6—H6⋯Cl1^ii^	0.93	2.72	3.384 (4)	129

Symmetry codes: (i) 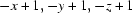; (ii) 
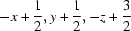
.

**Fig. 1.**
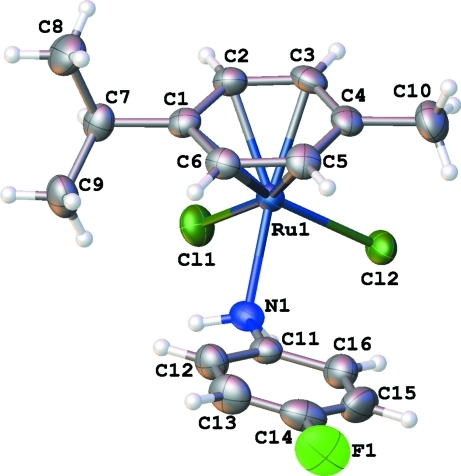
A thermal ellipsoid plot (50%) of the title compound showing the labeling scheme.

**Fig. 2.**
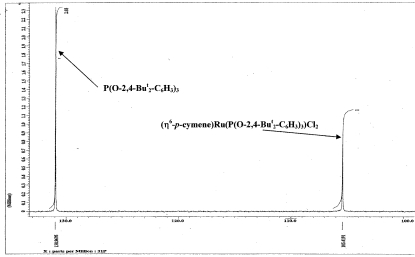
31P{1H} NMR Spectrum of the Equilibrium Mixture Prepared by Mixing Equimolar Amounts of (η6-p-cymene)Ru(4—F—C6H4NH2)Cl2 and P(O-2,4-But2-C6H3)3 in CDCl3 at 23 °C.

**Fig. 3.**
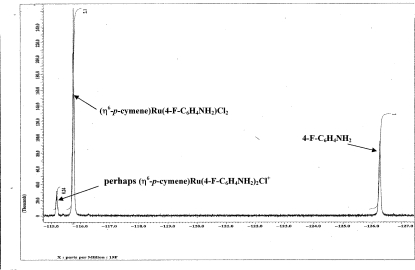
19F NMR Spectrum of the Equilibrium Mixture Prepared by Mixing Equimolar Amounts of (η6-p-cymene)Ru(4—F—C6H4NH2)Cl2 and P(O-2,4-But2-C6H3)3 in CDCl3 at 23 °C.

## supplementary crystallographic information

### Comment

The (η^6^-*p*-cymene)ruthenium(II)-dihalide motif has been used extensively for promotion of organic reactions (Boutadla *et al.*, 2010), bioinorganic studies (den Heeten *et al.*, 2010), and anti-tumor medical trials (Hanif *et al.*, 2010). Treating the commercially available dimer, di-*µ*-chloridobis-[chlorido(*p*-cymene)ruthenium(II)], with two molar equivalents of many neutral unidentate ligands (*L*) generates two moles of (η^6^-*p*-cymene)Ru(*L*)Cl_2_. The structures of several with aniline ligands (2,6-diisopropylphenyl, Burrell & Steedman, 1997; 4-chloro, Govindaswamy & Kollipara, 2006; 4-methyl, Begley, 1991) have been crystallographically determined. Structures of 4-fluoroaniline complexes have been reported for other late d-transition metals [palladium(II), Randell *et al.*, 2006; Padmanabhan *et al.*, 1985; nickel(II), Fawcett *et al.*, 2005].

Our interest in studying relative binding affinities of soft metal centers for ligands of moderate and weak donor power using ^19^F and ^31^P NMR spectroscopy (Hoffman *et al.*, 2009; Carter *et al.*, 2004) to monitor ligand-substitution equilibria led us to prepare the title complex. Single crystals were grown from vapor diffusion of heptane into a benzene solution of the 4-fluoroaniline complex. The nitrogen atom in the 4-fluoroaniline ligand is essentially coplanar with its aromatic ring, whose plane is oriented slightly down and away from the plane of the *p*-cymene ring. Structural parameters were similar to those reported for the other (η^6^-*p*-cymene)Ru(4—*X*—C_6_H_4_NH_2_)Cl_2_ piano-stool complexes above. The Ru—Cl, Ru—N, and Ru—C distances are quite ordinary. Somewhat greater differences exist between structural parameters of interest in the 4-fluoroaniline complexes of the divalent Pd and Ni moieties, likely because of their dissimilar combinations of d*^n^* configurations, coordination geometry, and ligand sets.

Our standard ligand-substitution reaction to determine relative affinities of neutral ligands (*L*) of moderate and weak donor power for (η^6^-*p*-cymene)RuCl_2_ employs the equilibrium below (eq. 1), where P* is the very sterically hindered triaryl phosphite, P(O-2,4-Bu*^t^*_2_-C_6_H_3_)_3_. Equilibrium constants are measured for different *L* employing

(η^6^-*p*-cymene)Ru(*L*)Cl_2_ + P* = (η^6^-*p*-cymene)Ru(P*)Cl_2_ + *L*              (1)

(i) the integrals of the respective ^31^P resonances for free P* and Cl_2_CymRu-P* and also (ii) the integrals of respective ^1^H resonances (for either free P*/Ru—P* or Cl_2_CymRu-*L*/Cl_2_CymRu-P*). For *L* = 4-fluoroaniline, the ^31^P-NMR spectrum of the equilibrium solution afforded by mixing equimolar amounts of (η^6^-*p*-cymene)Ru(4—F—C_6_H_4_NH_2_)Cl_2_ and P* in CDCl_3_ displayed just the two signals expected for free P* and Ru—P* (Fig. 2). However, the ^19^F NMR spectrum (Fig. 3) showed three resonances, a quick indication that our standard Cl_2_CymRu-*L* experimental design was invalid for use with anilines.

### Experimental

All solvents in synthesis were Fisher reagent-grade. To a stirred solution of 0.100 mmol [(η^6^-*p*-cymene)RuCl_2_]_2_ (Strem Chemicals) in 10 ml benzene in a 100-ml roundbottom flask was added 0.200 mmol neat 4-fluoroaniline (Sigma-Aldrich). Dripped slowly into the resulting dark-orange solution with stirring were 2.0 ml methyl *tert*-butyl ether and then 50 ml heptane. The yellow-orange crystals afforded were filtered and washed with two 5-ml portions of hexanes and air-dried (88% yield).

NMR analysis of this product in CDCl_3_ (Cambridge Laboratories) showed the following signals. ^1^H δ 0.21, 6H (d, ^3^J_H—H_=6.9 Hz); δ 2.11, 3H (*s*); δ 2.82, 1H (sept, ^3^J_H—H_=6.9 Hz); δ 4.90, 2H (*s*); δ 4.97, 2H (d, ^3^J_H—H_=6.1 Hz); δ 5.05, 2H (d, ^3^J_H—H_=6.0 Hz); δ 7.09, 2H (d of d; ^3^J_F—H_~^3^J_H—H_~ 8.5 Hz); δ 7.40, 2H (d of d; ^3^J_H—H_=8.5 Hz, ^4^J_F—H_=4.5 Hz). ^13^C{^1^H} δ 18.60(*s*), δ 22.06(*s*), δ 30.60(*s*), δ 79.65(*s*), δ 81.50(*s*), δ 95.91(*s*), δ 103.59(*s*), δ 116.32 (d, ^1^J_C—F_=22.5 Hz), δ 121.66 (d, ^2^J_C—F_=8.1 Hz), δ 141.30 (d, ^3^J_C—F_=1.7 Hz). ^19^F δ -115.71 (t of t, ^3^J_F—H_=8.5 Hz; ^4^J_F—H_=4.5 Hz); triplets overlap to form apparent "septuplet."

Suitable single crystals were grown from vapor diffusion of 30 ml heptane into a benzene solution of the 4-fluoroaniline complex (25 mg in 5 ml) over six days at room temperature. Traces of remaining liquid were removed by disposable glass pipet from the resulting red crystals which were washed twice with 5.0 ml hexanes and air-dried overnight in the dark.

### Refinement

Hydrogen atoms were placed in calculated positions and allowed to ride during subsequent refinement, with *U*_iso_(H) = 1.2*U*_eq_(C) and C—H distances of 0.93 Å for the aromatic H atoms, *U*_iso_(H) = 1.5*U*_eq_(C) and C—H distances of 0.96 Å for the methyl H atoms, *U*_iso_(H) = 1.2*U*_eq_(C) and a C—H distance of 0.98 Å for the methine H atom, and *U*_iso_(H) = 1.2*U*_eq_(N) and N—H distances of 0.90 Å for the amine H atoms.

### Crystal data

**Table:**

[RuCl_2_(C_10_H_14_)(C_6_H_6_FN)]	*F*(000) = 840
*M**_r_* = 417.30	*D*_x_ = 1.675 Mg m^−^^3^
Monoclinic, *P*2_1_/*n*	Mo *K*α radiation, λ = 0.71073 Å
Hall symbol: -P 2yn	Cell parameters from 25 reflections
*a* = 8.6492 (9) Å	θ = 8.5–13.2°
*b* = 12.2458 (13) Å	µ = 1.27 mm^−^^1^
*c* = 15.6471 (16) Å	*T* = 290 K
β = 93.271 (8)°	Prism, red
*V* = 1654.6 (3) Å^3^	0.26 × 0.25 × 0.20 mm
*Z* = 4	

### Data collection

**Table:**

Enraf–Nonius CAD-4 diffractometer	2284 reflections with *I* > 2σ(*I*)
Radiation source: fine-focus sealed tube	*R*_int_ = 0.048
graphite	θ_max_ = 25.4°, θ_min_ = 2.1°
θ/2θ scans	*h* = 0→10
Absorption correction: ψ scan (North *et al.*, 1968)	*k* = 0→14
*T*_min_ = 0.635, *T*_max_ = 0.779	*l* = −18→18
3234 measured reflections	3 standard reflections every 120 min
3027 independent reflections	

### Refinement

**Table:**

Refinement on *F*^2^	Primary atom site location: structure-invariant direct methods
Least-squares matrix: full	Secondary atom site location: difference Fourier map
*R*[*F*^2^ > 2σ(*F*^2^)] = 0.034	Hydrogen site location: inferred from neighbouring sites
*wR*(*F*^2^) = 0.090	H-atom parameters constrained
*S* = 1.00	*w* = 1/[σ^2^(*F*_o_^2^) + (0.0435*P*)^2^] where *P* = (*F*_o_^2^ + 2*F*_c_^2^)/3
3027 reflections	(Δ/σ)_max_ < 0.001
193 parameters	Δρ_max_ = 0.57 e Å^−^^3^
0 restraints	Δρ_min_ = −0.67 e Å^−^^3^

### Special details

**Table:**

Geometry. All e.s.d.'s (except the e.s.d. in the dihedral angle between two l.s. planes) are estimated using the full covariance matrix. The cell e.s.d.'s are taken into account individually in the estimation of e.s.d.'s in distances, angles and torsion angles; correlations between e.s.d.'s in cell parameters are only used when they are defined by crystal symmetry. An approximate (isotropic) treatment of cell e.s.d.'s is used for estimating e.s.d.'s involving l.s. planes.
Refinement. Refinement of *F*^2^ against ALL reflections. The weighted *R*-factor *wR* and goodness of fit *S* are based on *F*^2^, conventional *R*-factors *R* are based on *F*, with *F* set to zero for negative *F*^2^. The threshold expression of *F*^2^ > 2σ(*F*^2^) is used only for calculating *R*-factors(gt) *etc*. and is not relevant to the choice of reflections for refinement. *R*-factors based on *F*^2^ are statistically about twice as large as those based on *F*, and *R*- factors based on ALL data will be even larger.

### Fractional atomic coordinates and isotropic or equivalent isotropic displacement parameters (Å^2^)

**Table:**

	*x*	*y*	*z*	*U*_iso_*/*U*_eq_	
Ru1	0.22499 (3)	0.59291 (3)	0.611377 (18)	0.02585 (12)	
Cl1	0.24408 (14)	0.39815 (9)	0.63052 (7)	0.0442 (3)	
Cl2	0.29626 (13)	0.56893 (9)	0.46564 (6)	0.0391 (3)	
F1	0.8394 (4)	0.9472 (3)	0.6880 (2)	0.0811 (10)	
N1	0.4725 (4)	0.5795 (3)	0.6397 (2)	0.0335 (8)	
H1A	0.4864	0.5382	0.6871	0.040*	
H1B	0.5116	0.5417	0.5966	0.040*	
C1	0.0988 (5)	0.6207 (3)	0.7270 (2)	0.0322 (9)	
C2	−0.0055 (5)	0.5901 (4)	0.6567 (3)	0.0353 (9)	
H2	−0.0712	0.5309	0.6631	0.042*	
C3	−0.0120 (5)	0.6456 (4)	0.5792 (3)	0.0381 (10)	
H3	−0.0833	0.6247	0.5356	0.046*	
C4	0.0905 (5)	0.7347 (3)	0.5664 (3)	0.0359 (10)	
C5	0.1936 (5)	0.7656 (3)	0.6343 (3)	0.0377 (10)	
H5	0.2593	0.8247	0.6275	0.045*	
C6	0.2000 (5)	0.7087 (3)	0.7133 (2)	0.0337 (9)	
H6	0.2719	0.7295	0.7568	0.040*	
C7	0.1003 (5)	0.5586 (4)	0.8103 (2)	0.0375 (10)	
H7	0.0686	0.4832	0.7979	0.045*	
C8	−0.0172 (6)	0.6100 (5)	0.8675 (3)	0.0607 (15)	
H8A	−0.0165	0.5711	0.9208	0.091*	
H8B	−0.1187	0.6062	0.8395	0.091*	
H8C	0.0098	0.6850	0.8783	0.091*	
C9	0.2608 (6)	0.5567 (5)	0.8572 (3)	0.0513 (13)	
H9A	0.3347	0.5265	0.8203	0.077*	
H9B	0.2572	0.5127	0.9078	0.077*	
H9C	0.2908	0.6298	0.8729	0.077*	
C10	0.0887 (6)	0.7916 (4)	0.4821 (3)	0.0530 (13)	
H10A	0.0348	0.8598	0.4858	0.079*	
H10B	0.0372	0.7466	0.4390	0.079*	
H10C	0.1932	0.8050	0.4671	0.079*	
C11	0.5666 (4)	0.6756 (3)	0.6529 (2)	0.0312 (9)	
C12	0.6131 (5)	0.7101 (4)	0.7343 (3)	0.0406 (10)	
H12	0.5832	0.6705	0.7813	0.049*	
C13	0.7024 (6)	0.8016 (4)	0.7471 (3)	0.0510 (13)	
H13	0.7326	0.8251	0.8021	0.061*	
C14	0.7458 (5)	0.8573 (4)	0.6771 (4)	0.0508 (12)	
C15	0.7011 (5)	0.8263 (4)	0.5954 (3)	0.0539 (13)	
H15	0.7319	0.8665	0.5489	0.065*	
C16	0.6102 (5)	0.7351 (4)	0.5829 (3)	0.0424 (11)	
H16	0.5781	0.7133	0.5278	0.051*	

### Atomic displacement parameters (Å^2^)

**Table:**

	*U*^11^	*U*^22^	*U*^33^	*U*^12^	*U*^13^	*U*^23^
Ru1	0.02827 (18)	0.02503 (18)	0.02404 (17)	0.00145 (14)	−0.00045 (12)	−0.00363 (13)
Cl1	0.0591 (7)	0.0273 (5)	0.0457 (6)	0.0016 (5)	−0.0001 (5)	0.0026 (4)
Cl2	0.0435 (6)	0.0486 (7)	0.0252 (5)	0.0095 (5)	0.0027 (4)	−0.0046 (4)
F1	0.074 (2)	0.057 (2)	0.111 (3)	−0.0266 (18)	−0.011 (2)	0.001 (2)
N1	0.0335 (18)	0.035 (2)	0.0319 (17)	0.0073 (16)	0.0001 (14)	−0.0108 (15)
C1	0.032 (2)	0.034 (2)	0.031 (2)	0.0024 (17)	0.0060 (17)	−0.0075 (17)
C2	0.028 (2)	0.040 (2)	0.037 (2)	0.0000 (19)	0.0018 (16)	−0.0040 (19)
C3	0.029 (2)	0.045 (3)	0.040 (2)	0.013 (2)	−0.0053 (18)	−0.006 (2)
C4	0.036 (2)	0.034 (2)	0.038 (2)	0.0137 (19)	0.0031 (18)	0.0016 (18)
C5	0.046 (2)	0.026 (2)	0.041 (2)	0.0058 (19)	0.0048 (19)	−0.0049 (18)
C6	0.034 (2)	0.035 (2)	0.032 (2)	0.0024 (18)	−0.0010 (17)	−0.0111 (18)
C7	0.046 (3)	0.039 (2)	0.028 (2)	−0.007 (2)	0.0020 (18)	−0.0046 (18)
C8	0.062 (3)	0.082 (4)	0.039 (3)	0.008 (3)	0.013 (2)	−0.003 (3)
C9	0.059 (3)	0.057 (3)	0.037 (2)	−0.002 (3)	−0.007 (2)	0.007 (2)
C10	0.064 (3)	0.049 (3)	0.046 (3)	0.015 (3)	0.001 (2)	0.012 (2)
C11	0.026 (2)	0.036 (2)	0.032 (2)	0.0046 (18)	0.0015 (16)	−0.0056 (17)
C12	0.042 (2)	0.046 (3)	0.034 (2)	−0.001 (2)	−0.0034 (18)	−0.003 (2)
C13	0.051 (3)	0.056 (3)	0.044 (3)	−0.002 (3)	−0.008 (2)	−0.017 (2)
C14	0.035 (2)	0.042 (3)	0.074 (4)	0.003 (2)	−0.006 (2)	−0.007 (3)
C15	0.044 (3)	0.055 (3)	0.063 (3)	−0.003 (2)	0.013 (2)	0.015 (3)
C16	0.041 (2)	0.052 (3)	0.034 (2)	0.003 (2)	0.0025 (18)	−0.004 (2)

### Geometric parameters (Å, °)

**Table:**

Ru1—C2	2.154 (4)	C6—H6	0.9300
Ru1—C6	2.154 (4)	C7—C8	1.528 (6)
Ru1—C5	2.165 (4)	C7—C9	1.533 (6)
Ru1—N1	2.167 (3)	C7—H7	0.9800
Ru1—C3	2.180 (4)	C8—H8A	0.9600
Ru1—C4	2.184 (4)	C8—H8B	0.9600
Ru1—C1	2.192 (4)	C8—H8C	0.9600
Ru1—Cl1	2.4082 (11)	C9—H9A	0.9600
Ru1—Cl2	2.4138 (10)	C9—H9B	0.9600
F1—C14	1.372 (6)	C9—H9C	0.9600
N1—C11	1.439 (5)	C10—H10A	0.9600
N1—H1A	0.9000	C10—H10B	0.9600
N1—H1B	0.9000	C10—H10C	0.9600
C1—C6	1.412 (6)	C11—C12	1.381 (5)
C1—C2	1.433 (6)	C11—C16	1.384 (6)
C1—C7	1.508 (6)	C12—C13	1.369 (6)
C2—C3	1.388 (6)	C12—H12	0.9300
C2—H2	0.9300	C13—C14	1.361 (7)
C3—C4	1.427 (6)	C13—H13	0.9300
C3—H3	0.9300	C14—C15	1.367 (7)
C4—C5	1.401 (6)	C15—C16	1.374 (7)
C4—C10	1.491 (6)	C15—H15	0.9300
C5—C6	1.418 (6)	C16—H16	0.9300
C5—H5	0.9300		
			
C2—Ru1—C6	68.48 (15)	C5—C4—C3	118.3 (4)
C2—Ru1—C5	80.44 (17)	C5—C4—C10	121.3 (4)
C6—Ru1—C5	38.32 (16)	C3—C4—C10	120.4 (4)
C2—Ru1—N1	148.59 (14)	C5—C4—Ru1	70.5 (2)
C6—Ru1—N1	92.17 (14)	C3—C4—Ru1	70.8 (2)
C5—Ru1—N1	99.88 (15)	C10—C4—Ru1	129.4 (3)
C2—Ru1—C3	37.34 (16)	C4—C5—C6	121.4 (4)
C6—Ru1—C3	80.99 (16)	C4—C5—Ru1	72.0 (2)
C5—Ru1—C3	67.91 (17)	C6—C5—Ru1	70.4 (2)
N1—Ru1—C3	166.94 (15)	C4—C5—H5	119.3
C2—Ru1—C4	68.45 (16)	C6—C5—H5	119.3
C6—Ru1—C4	69.00 (15)	Ru1—C5—H5	131.2
C5—Ru1—C4	37.57 (16)	C1—C6—C5	120.9 (4)
N1—Ru1—C4	128.89 (15)	C1—C6—Ru1	72.5 (2)
C3—Ru1—C4	38.16 (16)	C5—C6—Ru1	71.2 (2)
C2—Ru1—C1	38.48 (15)	C1—C6—H6	119.5
C6—Ru1—C1	37.91 (15)	C5—C6—H6	119.5
C5—Ru1—C1	68.81 (16)	Ru1—C6—H6	129.1
N1—Ru1—C1	112.05 (14)	C1—C7—C8	109.0 (4)
C3—Ru1—C1	68.83 (15)	C1—C7—C9	112.6 (4)
C4—Ru1—C1	82.05 (15)	C8—C7—C9	109.9 (4)
C2—Ru1—Cl1	90.08 (12)	C1—C7—H7	108.4
C6—Ru1—Cl1	124.65 (12)	C8—C7—H7	108.4
C5—Ru1—Cl1	162.78 (12)	C9—C7—H7	108.4
N1—Ru1—Cl1	80.78 (9)	C7—C8—H8A	109.5
C3—Ru1—Cl1	112.26 (13)	C7—C8—H8B	109.5
C4—Ru1—Cl1	149.19 (12)	H8A—C8—H8B	109.5
C1—Ru1—Cl1	94.86 (11)	C7—C8—H8C	109.5
C2—Ru1—Cl2	126.86 (11)	H8A—C8—H8C	109.5
C6—Ru1—Cl2	145.24 (12)	H8B—C8—H8C	109.5
C5—Ru1—Cl2	108.47 (12)	C7—C9—H9A	109.5
N1—Ru1—Cl2	83.19 (9)	C7—C9—H9B	109.5
C3—Ru1—Cl2	96.05 (11)	H9A—C9—H9B	109.5
C4—Ru1—Cl2	87.26 (11)	C7—C9—H9C	109.5
C1—Ru1—Cl2	164.70 (11)	H9A—C9—H9C	109.5
Cl1—Ru1—Cl2	88.72 (4)	H9B—C9—H9C	109.5
C11—N1—Ru1	120.8 (2)	C4—C10—H10A	109.5
C11—N1—H1A	107.1	C4—C10—H10B	109.5
Ru1—N1—H1A	107.1	H10A—C10—H10B	109.5
C11—N1—H1B	107.1	C4—C10—H10C	109.5
Ru1—N1—H1B	107.1	H10A—C10—H10C	109.5
H1A—N1—H1B	106.8	H10B—C10—H10C	109.5
C6—C1—C2	116.8 (4)	C12—C11—C16	119.4 (4)
C6—C1—C7	122.7 (4)	C12—C11—N1	121.0 (4)
C2—C1—C7	120.4 (4)	C16—C11—N1	119.6 (4)
C6—C1—Ru1	69.6 (2)	C13—C12—C11	121.1 (4)
C2—C1—Ru1	69.3 (2)	C13—C12—H12	119.4
C7—C1—Ru1	130.9 (3)	C11—C12—H12	119.4
C3—C2—C1	122.4 (4)	C14—C13—C12	118.1 (4)
C3—C2—Ru1	72.4 (2)	C14—C13—H13	120.9
C1—C2—Ru1	72.2 (2)	C12—C13—H13	120.9
C3—C2—H2	118.8	C13—C14—C15	122.5 (5)
C1—C2—H2	118.8	C13—C14—F1	119.3 (5)
Ru1—C2—H2	129.1	C15—C14—F1	118.2 (5)
C2—C3—C4	120.2 (4)	C14—C15—C16	119.2 (5)
C2—C3—Ru1	70.3 (2)	C14—C15—H15	120.4
C4—C3—Ru1	71.1 (2)	C16—C15—H15	120.4
C2—C3—H3	119.9	C15—C16—C11	119.6 (4)
C4—C3—H3	119.9	C15—C16—H16	120.2
Ru1—C3—H3	131.6	C11—C16—H16	120.2

### Hydrogen-bond geometry (Å, °)

**Table:**

*D*—H···*A*	*D*—H	H···*A*	*D*···*A*	*D*—H···*A*
N1—H1B···Cl2^i^	0.90	2.39	3.225 (3)	154
C6—H6···Cl1^ii^	0.93	2.72	3.384 (4)	129

Symmetry codes: (i) −*x*+1, −*y*+1, −*z*+1; (ii) −*x*+1/2, *y*+1/2, −*z*+3/2.
